# Urgent Response Needed: Addressing the Dengue Crisis in the Andean and Southern Cone Latin American Regions

**DOI:** 10.4269/ajtmh.24-0178

**Published:** 2024-08-06

**Authors:** Esteban Ortiz-Prado, Juan S. Izquierdo-Condoy, Jorge Vásconez-González

**Affiliations:** One Health Research Group, Faculty of Health Science, Universidad de Las Americas, Quito, Ecuador

## Abstract

The dengue crisis in the Latin American region is currently intensifying, exacerbated by heavy rains, widespread flooding, and the onset of the El Niño–Southern Oscillation. The indirect consequences of the COVID-19 pandemic, which weakened healthcare systems, have further compounded the situation. Comparing the first 15 weeks of 2023 with the same period in 2024, we observed a significant average increase of 600% in the number of new cases. This translates to a 536% rise in the composite rate per 100,000 inhabitants across all countries. Brazil experienced a staggering surge from 1,425,000 cases in the initial 15 weeks of 2023 to 5,177,989 cases in the corresponding period of 2024. Similarly, Paraguay witnessed a notable escalation, with cases soaring from 12,497 in 2023 to more than 240,000 thus far in 2024, marking an increase of more than 1,825%. Bolivia, however, witnessed a reduction in cases, though the cause remains unclear. Urgent action is imperative to address this escalating crisis. Strengthening surveillance systems, enhancing vector control programs, and implementing effective public health campaigns are critical. Immediate and coordinated action by regional governments and health authorities is essential to mitigate the growing dengue crisis and safeguard public health in the region.

## INTRODUCTION

In Latin America, political and social problems are constant, as are health issues. After the arrival of COVID-19, figures showed how a region with a high degree of inequality and health systems of low resilience provided the perfect mix to suffer one of the strongest impacts in terms of morbidity and mortality attributed to COVID-19.^1^ At that time, efforts to contain the virus’s arrival meant that control strategies for other endemic diseases, such as dengue, malaria, and chikungunya, among others, were relegated to secondary priority.^2^ Today, 4 years after the arrival of SARS-CoV-2 in the region, the consequences of neglect in terms of epidemiological surveillance, resource allocation, prevention, and outbreak preparedness combined with the occurrence of climatological phenomena such as the El Niño–Southern Oscillation (ENSO) are causing rapid, progressive, and lethal increases in terms of dengue incidence, mortality, and burden of diseases.^3^^,^^4^

Dengue, as the most prevalent arbovirus, presents a significant threat to the uniquely unequal, impoverished, and migrant-heavy Latin American region. Although cyclic, weather-driven mosquito-transmitted epidemics typically occur globally every 3–5 years; this year the influence of ENSO and floodings in the Amazon Basin have exacerbated the situation, resulting in a notable surge in cases. Compared with 2023, the Americas have witnessed an alarming increase of more than 600% in new cases, representing more than 80% of the global burden (Table 1).^5^^,^^6^

**Table 1:** Comparison of the number of dengue fever cases and rates per 100,000 inhabitants during the first 15 epidemiological weeks of 2023 and 2024 in Southern Cone Countries of the Americas

Country	2023		2024		Paired *t*-Test *P*-Value	% Change Cases	% Change Rate
	Total Number of Cases (15 weeks)	Average Rate per 100k (15 weeks)	Total Number of Cases (15 weeks)	Average Rate per 100k (15 Weeks)			
Ecuador	5,850	2.1	21,651	7.9	<0.0001	270%	267%
Colombia	12,497	1.8	103,323	9.3	<0.0001	727%	407%
Peru	42,511	8.2	160,757	30.9	0.0004	278%	275%
Argentina	51,619	10.3	315,942	46.3	0.0035	512%	351%
Bolivia	124,657	67.1	25,364	13.5	<0.0001	−80%	−80%
Brazil	1,425,384	43.9	5,177,989	158.6	<0.0001	263%	261%
Uruguay	23	0.0	254	0.5	0.0001	1,004%	1,004%
Paraguay	12,497	12.1	240,518	231.2	<0.0001	1,825%	1,804%
Total	1,675,038	18	6,045,798	62	N/A	600%	536%

N/A = not applicable.

According to data from the Pan American Health Organization (PAHO) for 2023 and 2024, countries in the sub-Andean region and the Southern Cone have experienced a concerning increase in the number of new cases compared with previous years (Table 1).^7^

**Figure 1.**
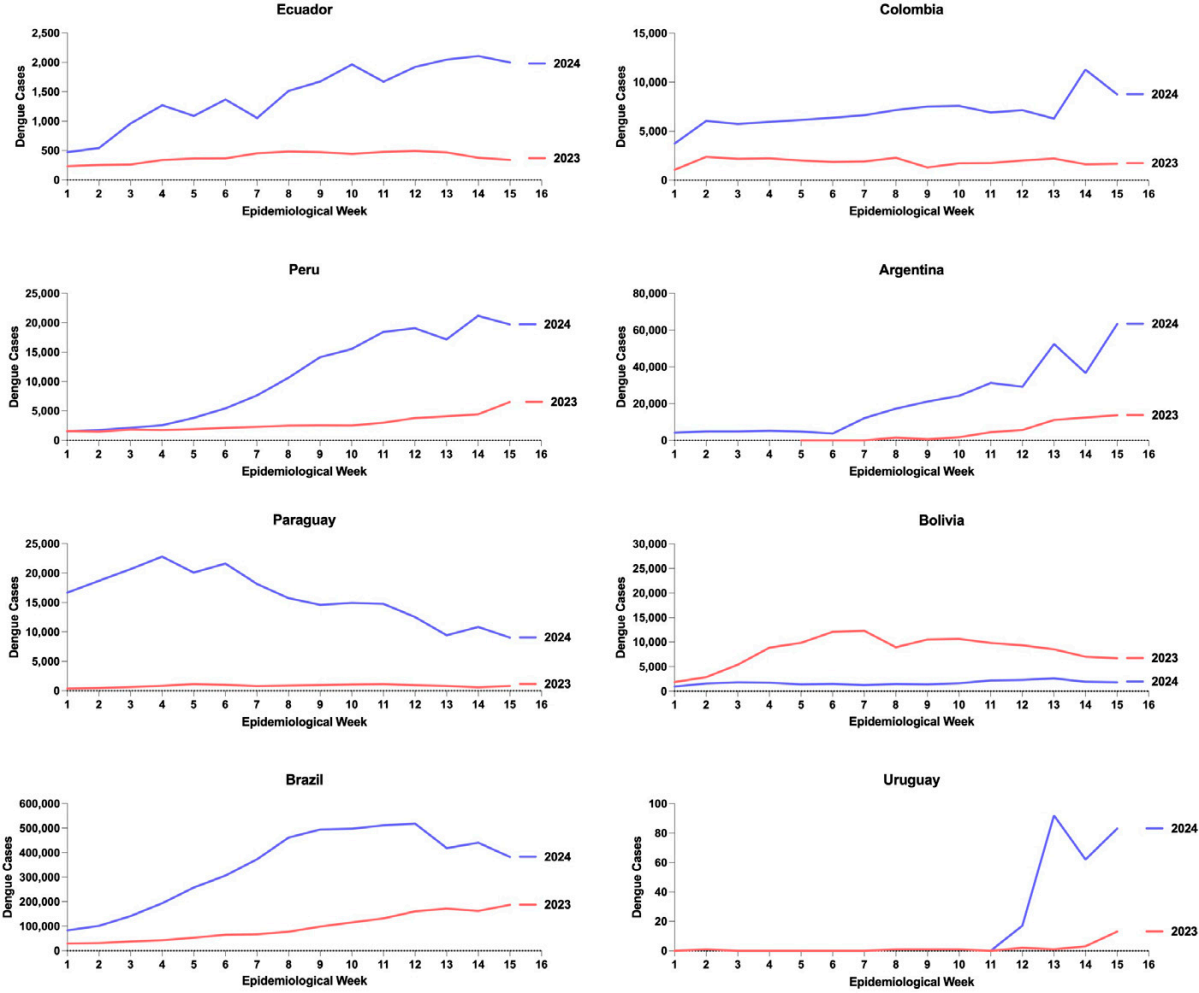
Epidemiological Weeks 1–15 dengue trends: Increase of dengue cases in the countries of the Andean region and the Southern Cone from 2023 to 2024.

In the Andean region, we observed that Ecuador experienced a more gradual increase in new cases from epidemiological week (EW) 1 to EW 15 compared with some of the peaks seen (e.g., in EW 7 and EW 8 and EW 14 and EW 15 in Colombia). Conversely, Peru showed a sharp, rapid increase in cases starting from EW 4, seemingly decreasing only around EW 14. In Argentina, 2024’s EW 6 marked a dramatic increase in cases. Paraguay, despite a striking increase in cases compared with 2023, began to decrease from EW 4. Brazil, on the other hand, has exhibited a steady increase from EW 1, peaking around EW 12 and gradually decreasing by EW 15. Uruguay, with minimal cases, has shown a noteworthy increase (Figure 1).

**Figure 2.**
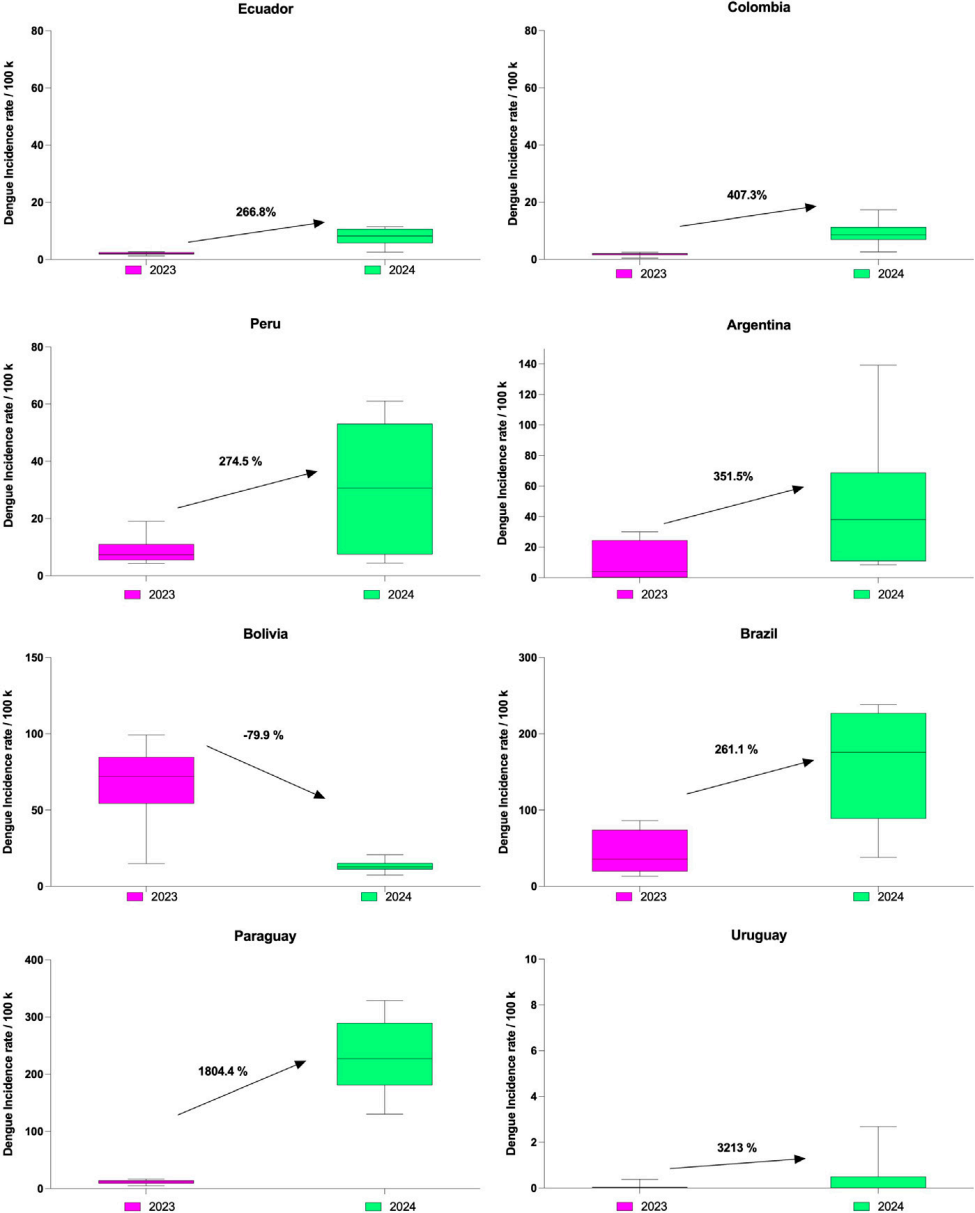
Epidemiological Weeks 1–15 dengue trends: Increase of dengue incidence rate per 100,000 in the countries of the Andean region and the Southern Cone from 2023 to 2024. The error bars represent data variability, showing the spread and outliers in dengue incidence rates between 2023 and 2024.

Analyzing cases per 100,000 inhabitants, comparing the average rate of the 15 EWs from 2023 versus the same EWs of 2024, we found that Uruguay saw a significant 3,213% increase from 2023. Paraguay followed with an abrupt 1,800% increase, whereas Brazil, Peru, and Ecuador showed similar average increases of around 250–280% from 2023 to 2024. Colombia and Argentina exhibited dramatic increases of 407% and 351%, respectively, whereas Bolivia experienced a 79% decrease compared with 2023, with fewer cases in 2024 (Figure 2).

This scenario occurred in the entire region, where an increase of more than 570% in dengue incidence rate has been reported compared with previous years, resulting in more than 17,000 deaths to date within the region.^8^ For the year 2023, the mortality rate in Andean countries (Bolivia, Colombia, Ecuador, and Peru) reached 8.8%, with more than 9,414 severe cases of dengue, accounting for 0.6% of total cases. By 2024, in the Andean region, there were 11,400 severe cases of dengue with a mortality rate of 5.7%, slightly lower than the previous year, but the proportion of severe dengue cases remains at 0.6%. In contrast, in the Southern Cone, which includes Argentina, Brazil, Chile, Paraguay, and Uruguay, the mortality rate in 2023 was 3.5%, with more than 4,554 severe cases, representing 0.1% of total cases. However, by 2024, there were 16,332 deaths in the Southern Cone, accounting for a mortality rate of 4.7%, and more than 21,560 severe cases, also representing 0.1% of the total cases. These findings suggest that there may be underdiagnosis in the Andean region, leading to seemingly higher mortality rates, but they also indicate an increase and crisis in 2024.^9^

According to data from the Health Information Platform for the Americas (PLISA) and PAHO regarding dengue, the region is witnessing not only a significant increase in the overall number of cases but also an apparent rise in the proportion of severe cases and fatalities, particularly within the Andean region. This trend in relation to the total number of cases suggests an escalation in the severity of outbreaks. In contrast, such trends are not as pronounced in Southern Cone countries, likely because of their more advanced diagnostic capabilities.^9^

These concerning trends not only challenge epidemic responses in affected countries but also expose vulnerabilities in resilient health systems and inadequate surveillance strategies, reflecting a reactive rather than proactive approach. It is alarming to witness that, despite experiencing a pandemic of unprecedented scale, health systems are once again being marginalized, with other political agendas taking precedence and basic expected responses being neglected. This negligence leads to health crises that further compound regional challenges.

Addressing these issues requires a concerted and urgent effort from governments and health authorities to bolster surveillance and control programs for vector-borne diseases. Moreover, there is a pressing need to enhance prevention measures and establish more effective health promotion mechanisms. In addition, we acknowledge the importance of reorganizing health services and optimizing care provision to dengue patients. Training clinical personnel in early case identification and warning signs, along with implementing well-established clinical care pathways, can significantly mitigate the impact of dengue in terms of morbidity and mortality.^10^ Furthermore, it is crucial to incorporate epidemiological alerts and emergency declarations into our strategies. For example, the declaration of an emergency in 20 of 24 regions in Peru, along with PAHO and WHO alerts, highlights the severity of the situation and plays a pivotal role in enhancing public awareness and mobilizing resources. These measures have been instrumental in improving risk communication and enhancing the public’s responsiveness to prevention campaigns. Therefore, integrating these interventions into the current context is imperative to effectively address the dengue crisis and safeguard public health.

This perception piece aims to raise awareness and encourage regional governments to take immediate yet enduring actions to change the paradigm in which we currently operate. We often respond to health crises as they occur rather than preventing them proactively, and sometimes resources are prioritized for certain areas to the detriment of others. Key areas identified as lacking and requiring action include the collection and sharing of regional systemic data in real time, strengthening, creating, or enhancing vector control programs. Such programs should include education to prevent the emergence of disease-transmitting mosquitoes, the elimination of aquatic breeding sites where mosquitoes can proliferate, resumption of vector control campaigns using insecticides in areas of higher contamination, introduction of programs for the distribution of bed nets or mosquito nets, and reinforcing the importance of providing mosquito repellent in vulnerable areas.^11^^,^^12^ With the arrival of “El Niño,” which has been milder than previously projected, it is crucial to develop and implement effective response strategies to mitigate the further impact of the outbreak. Considering that there are 37 EWs left in the year, timely and considered actions are essential to prevent worsening conditions.

Furthermore, this crisis should advocate for the initiation of comprehensive public awareness campaigns. These campaigns should be meticulously crafted to educate communities about prevention strategies, recognition of symptoms, and the crucial importance of seeking immediate medical attention to mitigate the effects of the disease.

The urgency to act constitutes a pivotal call to action. Ignoring the alarming increase in cases will only result in more individuals falling ill, diminishing surveillance responses, and exacerbating resource scarcity. Such inaction will inevitably lead to an escalation of the already severe dengue crisis. Immediate and decisive measures are imperative to prevent further deterioration of public health and to ensure the well-being of affected populations.
